# Achieving optimal SERS through enhanced experimental design

**DOI:** 10.1002/jrs.4855

**Published:** 2015-12-16

**Authors:** Heidi Fisk, Chloe Westley, Nicholas J. Turner, Royston Goodacre

**Affiliations:** ^1^ School of Chemistry, Manchester Institute of BiotechnologyUniversity of Manchester131 Princess StreetManchesterM1 7DNUK

**Keywords:** SERS, chemometrics, optimisation, design of experiment, genetic algorithm

## Abstract

One of the current limitations surrounding surface‐enhanced Raman scattering (SERS) is the perceived lack of reproducibility. SERS is indeed challenging, and for analyte detection, it is vital that the analyte interacts with the metal surface. However, as this is analyte dependent, there is not a single set of SERS conditions that are universal. This means that experimental optimisation for optimum SERS response is vital. Most researchers optimise one factor at a time, where a single parameter is altered first before going onto optimise the next. This is a very inefficient way of searching the experimental landscape. In this review, we explore the use of more powerful multivariate approaches to SERS experimental optimisation based on design of experiments and evolutionary computational methods. We particularly focus on colloidal‐based SERS rather than thin film preparations as a result of their popularity. © 2015 The Authors. Journal of Raman Spectroscopy published by John Wiley & Sons, Ltd.

## Introduction

Surface‐enhanced Raman scattering (SERS) is a vibrational spectroscopic technique that employs the use of roughened metal substrates, giving rise to large enhancements of the Raman signal and overcoming the inherent weakness of the traditional technique. Typically, enhancements in the range of 10^4^–10^6^ can be observed as well as reports of single molecule detection.^1^, ^2^, ^3^ Additional enhancement may be observed when the SERS technique is coupled with resonance Raman, a technique referred to as surface‐enhanced resonance Raman scattering. The SERS phenomenon was first observed by Fleischmann *et al*. in 1974, and these authors observed interactions of pyridine at the surface of a roughened silver electrode, which led to a substantial increase in Raman intensity, which at that time could not be explained.^4^

Despite SERS being discovered over 40 years ago, the mechanism of enhancement is still under debate within the SERS community. It is thought that there are two principal mechanisms that give rise to the dramatic enhancement: the electromagnetic (EM) and chemical transfer mechanisms. The first theory, thought to be the more dominant,^5^ occurs because of an interaction between the analyte and the plasmon excitation on the roughened metal surface. The incident laser light excites coherent wave oscillations of the surface electrons in the metal, called a localised surface plasmon. These oscillations at the nanostructured surface result in amplification of EM fields, which can reach out to the analyte located in close proximity.^6^, ^7^ The second theory proposes that the analyte forms a chemical bond to the metal surface and excitation occurs via the transfer of electrons from the metal to the analyte and *vice versa*, generating a charge‐transfer complex thus increasing the molecular polarisability.^8^

The SERS technique has become increasingly popular in a wide variety of research fields because of its rapid, non‐destructive and label‐free nature, whilst generating highly specific structural information. Examples of these broad applications include biosensors,^9^ detection of illicit drugs,^10^ identification of DNA bases^11^ and the discrimination of bacteria.^12^

Despite the increasing number of SERS applications, a common limitation, and real bottleneck within the community, is the difficulty in generating reproducible spectra and consistent enhancements within and between different experiments.^13^, ^14^, ^15^, ^16^ Moreover, the literature can be confusing because of the many different substrates and conditions used: although this can be advantageous (due to the myriad of different SERS‐active substrates and their varying properties), there tends to be an over‐reliance on specific conditions meaning suboptimal enhancements are frequent. Consequently, the need to optimise the system to obtain optimum SERS responses (for each specific analyte) is an essential step in SERS experiments.

## Parameters to consider in optimisation

**Figure 1:**
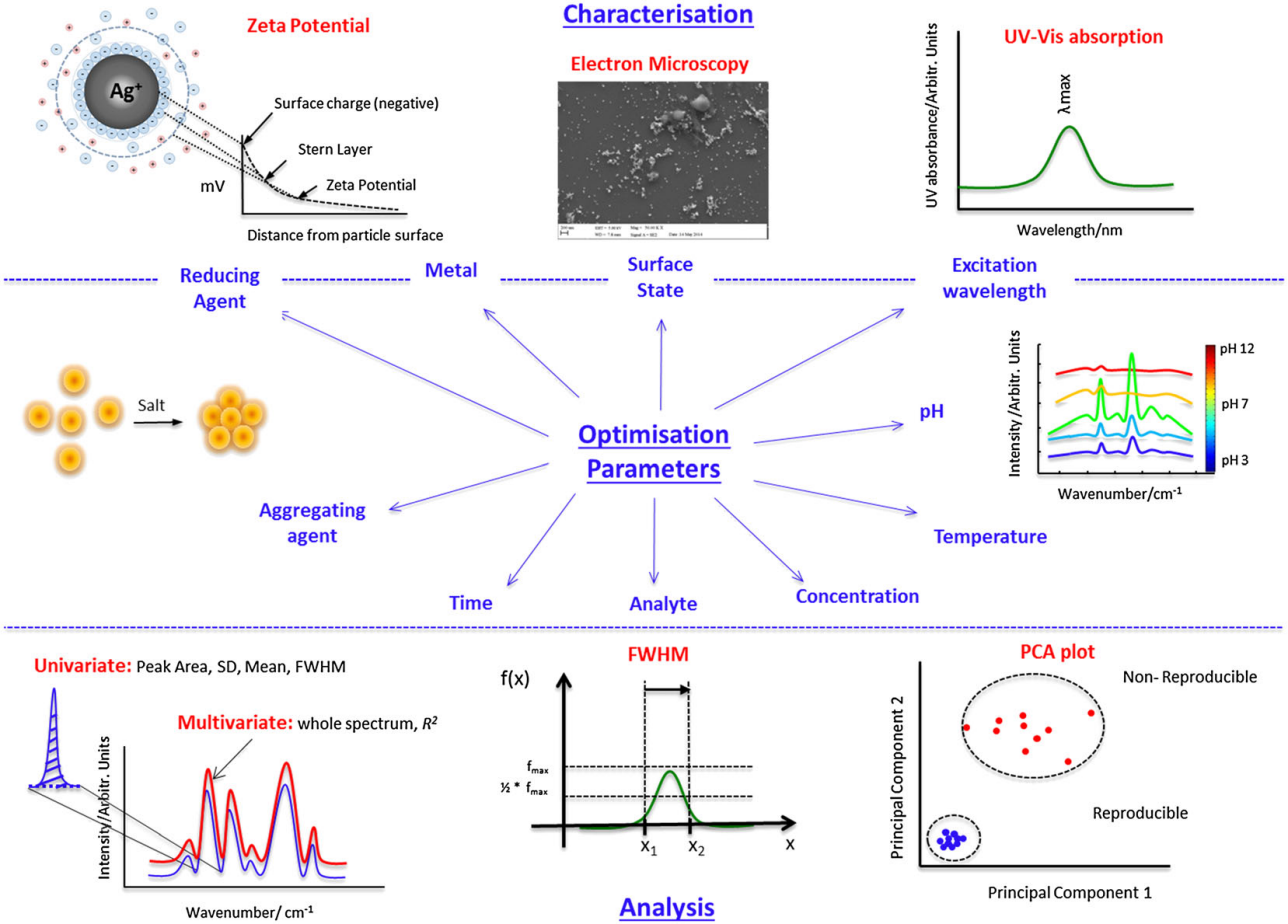
Schematic depicting which variables should be considered when optimising a system for achieving the best surface‐enhanced Raman scattering response in terms of the signal enhancement being strong, robust and reproducible. This schematic highlights the individual parameters, various characterisation techniques as well as the data analysis approach that need to be considered during the design of experiments phase. FWHM, full width half maximum; PCA, principal component analysis; SD, standard deviation.

Central to SERS is the production of a surface that is nanoscale in roughness, produced in a reproducible way, and thus, it is important that several batches of the same substrate are produced during the optimisation process. There are several parameters that need to be carefully considered, as well as various characterisation techniques that can be used to assess the suitability of these conditions (Fig. 1). To note, although the optimisation parameters we discuss in this review are more applicable for colloidal solutions, the same theory applies for thin films (with a different parameter set employed).

The selection of laser excitation wavelength has a significant impact on experimental capabilities, and whilst typically visible excitation is used (e.g. 488, 514.5, 532 and 633 nm),^17^, ^18^ SERS has been reported from the near UV (325 nm),^19^ the near IR (785 and 830 nm) as well as recently at even longer wavelengths such as 1064, 1280 and 1550 nm.^20^, ^21^, ^22^, ^23^ This selection process is often determined by a need to compromise between minimising sample fluorescence and maximising scattering efficiencies (especially if resonance is also used). Most biological samples are fluorescent in nature; therefore, choosing high frequency lasers (high power) would be deemed appropriate, although there is evidence that the metal particles can reduce the level of fluorescence.^24^, ^25^

A requirement for SERS is a metal surface with nanoscale roughness (normally in the range 5–100 nm), and typically, SERS substrates are in two forms: either solid state or in a colloidal suspension.^26^ Solid state SERS involves a flat surface with a roughened metal layer on top. They are usually quite expensive and more difficult to produce, as well as being site specific, and consequently, there is less control in terms of optimisation. On the other hand, colloids involve metal nanoparticles suspended in solution and are generally the more favoured substrate due to their low cost and ease of preparation. Various reducing agents are available (such as sodium citrate and hydrochloride hydroxylamine), which reduce the metal and control the nanoparticle size.^27^, ^28^

Most SERS substrates consist of the coinage metals, predominantly gold and silver, as their surface plasmons lie in the visible region of the EM spectrum and thus coincide with common Raman excitation wavelengths.^18^ Generally, analytes that contain thiol groups will exhibit much stronger binding to gold surfaces, whereas analytes that contain amine groups tend to have a higher affinity for silver.^29^ Manipulation of the analyte/surface binding can be achieved through varying the pH either of the analyte itself or the surface charge (through choice of reducing agent as well as charge neutralising chemicals such as poly‐Lysine or spermine).

Evidently, small changes in the metal particles surface charge can have serious implications in terms of stability, sensitivity to the environment as well as electro‐kinetic properties. The nanoparticles surface charge is of the utmost importance as interaction between analyte molecules and colloidal particles is a primary requisite for obtaining strong surface enhancement. When the analyte and surface have the same charge, the adsorption process can be strongly hindered, and if the colloidal particles fail to exceed a minimum repulsion with one another, they will aggregate and precipitate out of solution.^30^ In order to make the system more acidic (i.e. protonation of analyte) HCl or citric acid is typically added. In contrast, to deprotonate an analyte and make a more basic environment, NaOH is commonly used. Michota *et al*. (2003) and Alharbi *et al*. (2014) have demonstrated how the different binding modes are dependent on pH in relation to 4‐mercaptobenzoic acid and nicotine, respectively.^31^, ^32^

Employing an aggregating agent (common examples include NaCl and KNO_3_) can also have a major influence on the SERS response and so careful consideration and screening is often required. Aggregating agents are commonly employed to aggregate the different, irregular nanoparticles together. This leads to increased interactions between the colloid particles, affording larger surface plasmon resonances and greater surface enhancement. However, if too much aggregating agent is added, the colloid particles will quickly precipitate out of solution and the SERS signal will be lost.^16^, ^33^

Lastly, perhaps key to improving the reproducibility and often overlooked in experiments is the time allowed for optimal aggregation to occur; i.e. when do you get the most stable SERS responses and this is often analyte/system dependent. Therefore, time studies should be performed for each SERS experiment.

## Characterisation techniques for nanoparticle synthesis

In order to establish the optimum conditions, especially when initial decisions regarding substrate and metal are key, various techniques are typically employed by researchers.

After synthesising the various colloids available, the first initial step is to measure its UV–Vis absorption (Fig. 1). The *λ*_max_ of silver colloids is around 400 and 520 nm for gold colloids (with specific surface plasmon bands available for differently reduced colloids), and comparisons with the literature are often made, rather than further characterisation (e.g. electron microscopy; EM).^27^, ^28^ The *λ*_max_ full width half maximum (FWHM) of the prepared colloid allows for assessment of the nanoparticle size distribution: a narrower peak is indicative of a more monodisperse and reproducible colloid. A combination of both values should be used to establish which batch of colloid is selected for further experimental studies, although we note that this alone does not guarantee SERS for the analyte of interest.

In addition, further characterisation techniques can be employed such as EM and zeta potential analysis. EM is used to determine the size, morphology and distribution of the nanoparticles – and commonly, formation of agglomerated nanoparticles occurs more readily with silver substrates than gold. The zeta potential provides useful information regarding the charge carried by the nanoparticles and therefore the stability and ability to interact with analyte molecules. Larmour *et al*. (2012) explains that a colloid is considered stable if the zeta potential value is less than −30 mV or greater than +30 mV – with citrate‐reduced, hydroxylamine‐reduced and ethylenediaminetetraacetic acid‐reduced metal ions considered the most stable. Borohydrydride‐reduced silver colloids are notoriously unstable with a very high zeta potential of around −7.8 mV.^34^

## Data analysis methods and characterisation techniques

There are various data analysis methods that can be employed in order to establish the suitability and reproducibility of a set of conditions. The most simple techniques involve univariate analysis – whereby the peak area of a characteristic vibration is plotted against a certain parameter, e.g. concentration and time point. Normally, other statistical assessments are calculated such as the mean and standard deviation, with the latter describing the associated errors. The FWHM can also be used to evaluate how suitable conditions are, with sharper peaks (reflected by a reduced FWHM) more favourable. In addition, it is believed that the stronger, characteristic and narrow peaks readily allow multiplexing whereby several analytes are detected simultaneously.^32^, ^35^, ^36^

Although currently less common, multivariate analysis can be employed whereby the whole SERS spectrum is considered in the assessment process. One approach is to consider how well correlated repeat experiments of the same conditions are. Here, correlation coefficients are used and *R^2^* values closer to 1 indicate a good fit. As well as this, various chemometric approaches can be applied to simplify complex multivariate SERS data for interpretation and analysis, such as the use of principal component analysis (PCA). PCA is a widely used, unsupervised method, i.e. it does not require *a priori* information, representing the natural variance within a data set. Tight clustering of replicate measurements in PCA scores space (Fig. 1) indicates that the spectra are more similar and thus the conditions are more reproducible. Feng, Webster and Mabbott present good examples of how PCA has been used on Raman data.^37^, ^38^, ^39^

Combining all these characterisation techniques illustrated in Fig. 1, with the various data analysis methods available, should help determine which colloid is most suitable to use, especially in terms of batch‐to‐batch variation. Moreover, we advocate that multiple batches of the same metal substrate are always produced so that adequate statistics can be generated; far too often, a single experiment is reported, which may be atypical.

## Design of experiment

Most researchers require suitably characterised and optimised protocols and instruments for their research purposes with a particular focus on increased sensitivity, resolution, reproducibility and lower limits of detection and quantification. Optimisation usually involves conducting appropriate experiments that will provide data on one or more performance criteria under a variety of conditions. Commonly, researchers employ the one factor at a time principle, where a single parameter is optimised first before going onto optimise the next, and so on and so forth.^40^ However, whilst relying on the knowledge and skill of the researcher, a major flaw in this approach is that this assumes a lack of statistical interaction of the parameters. For many cases, parameters within an experiment are interdependent thus contributing to a joint effect, meaning this approach rarely provides a definitive solution.

A similar problem, and mentioned earlier, is the dependence on certain conditions when conducting different experiments. It is common for researchers to focus on using a set of conditions that are familiar or those that they have had success with, rather than exploring other alternatives. A common starting point that seems to be adopted is to replicate ‘optimal’ conditions presented by others and switch to a one factor at a time/‘trial and error’ approach if and when problems arise. By contrast, we believe that a more elegant approach, perhaps most effective of all, and often overlooked, is to perform a systematic design of experiment (DoE) prior to research.

Design of experiment is a well‐established proven statistical method first pioneered in the 1920s by R.A. Fisher.^41^ This statistical design of experiments uses replication, blocking, randomisation and orthogonality to recognise the statistical interaction of variables and employs statistics as an objective means of drawing conclusions. These general principles of experimental design are described in detail by Morgan, Underwood, Quinn and Keough and indeed any standard statistical textbook.^42^, ^43^, ^44^

There are different levels of design that can be applied depending on the nature of the experiment. In its simplest form, a fractional factorial design can be employed – whereby experiments are performed in order to identify which factors are the most critical. This approach is often used at the beginning of an optimisation project where many factors are likely to have little or no effect on the response (SERS enhancement). Conversely, a full factorial design combines all possible combinations of factors, affording the identification of significant interactions between them, but can be more time consuming.^45^ For example, if one wanted to optimise a modest eight parameters (e.g. type of metal, reducing agent, aggregating agent, volumes, concentrations and time of acquisition), and each of these could take one of just ten values, then the number of possible experiments is 8^10^ or just over 10^9^! Clearly, an exhaustive search of all possible experiments is not plausible.

The common phases identified in any DoE approach include^46^

- First, the identification of factors that may affect the outcome of the experiment and a response that can give an objective measure of this outcome. For example, this could be any optimisation parameter discussed in Fig. 1 and the effect this has on the intensity of the SERS signal (response).
- Next, is the choice of an appropriate experimental design – for example, either a full factorial design or a fractional factorial design – this will result in the generation of the design matrix, which aims to carefully select which experimental conditions need to be conducted. This small fraction of selected experiments is expected to be sufficient to reveal the most important features of the problem studied.
- These conditions are then performed in the lab and assessed by various data analysis methods, i.e. peak area(s), standard deviation, mean and PCA, as described earlier. Ultimately, whichever data analysis method is selected, the plots should describe the same trends in the results, enabling one to draw conclusions and plan the next step(s) to be taken.

**Figure 2:**
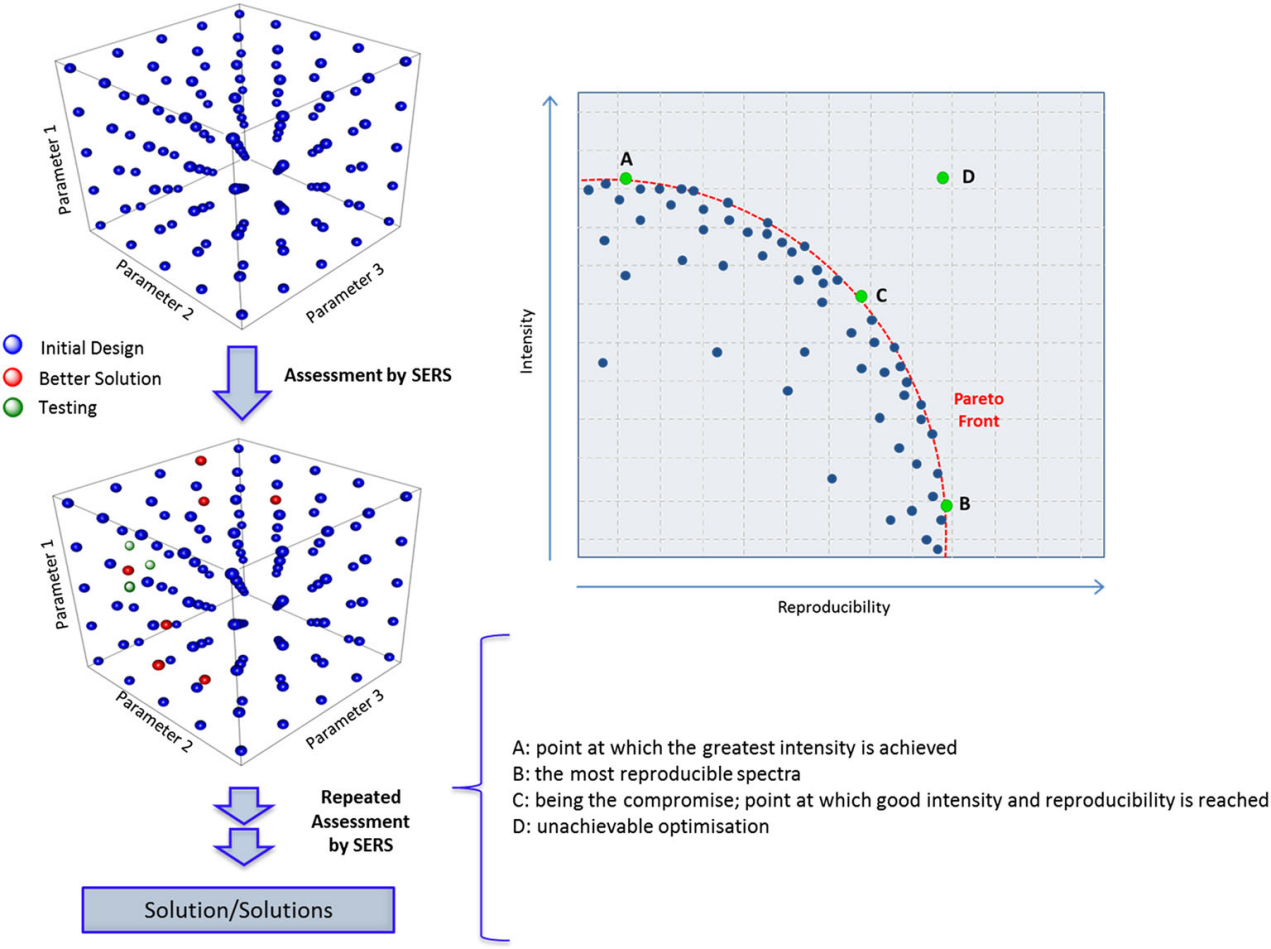
A 3D representation of a typical design of experiment that incorporates different parameters that needs to be optimised in order to achieve the most optimal surface‐enhanced Raman scattering (SERS) response. Sequential rounds of assessment are performed until a realistic solution(s) is obtained. The concept of Pareto optimality is demonstrated: when optimising parameters within a SERS experiment, it is not necessarily true that the conditions optimal to signal enhancement are also optimal to reproducibility and so a trade‐off between the two objectives must be established. As an example, the parameters to be optimised concurrently include parameter 1 = pH, parameter 2 = concentration of colloid and parameter 3 = aggregating agent.

A generic example of this process is illustrated in Fig. 2. A fractional factorial design has been generated to find the best solution(s) in the optimisation of a specific analyte for optimal SERS enhancement. The design is for a set of experiments in which three factors are thought to be important (these could for example be pH, concentration and aggregating agent) and is modelled on a cube to represent the experimental region being explored. The blue circles represent the initial experiments to be conducted and analysed using SERS, and these have been chosen to span the experimental search space adequately. After SERS assessment (and data analysis), certain sets of conditions are identified as the best solutions (denoted by the red spheres) and so more experiments based on these conditions are tested (denoted by the green spheres) and analysed to find the most optimum solution(s). This iterative process should determine which sets of factors are important with the number of experiments conducted significantly reduced.

It should be noted that when optimising parameters within a SERS experiment, it is not necessarily true that the conditions optimal to signal enhancement are also optimal to reproducibility and so a trade‐off between the two objectives must be established. This behaviour is an example of the Pareto principal. If one set of conditions leads to greater enhancement but lower reproducibility (or *vice versa*) than another set, one cannot assume that either set is superior. The sets of solutions within the entire search space (i.e. all combinations of parameters) that are not dominated by other solutions are termed the Pareto optimal front (denoted by the dashed arc in Fig. 2).^47^

There are few SERS examples that have utilised the idea of DoE, with Mabbott and colleagues (2013) providing a key example in the optimisation of mephedrone detection. In this optimisation, the number of statistically significant experiments was greatly reduced from 1722 to 288 by adopting the fractional factorial approach and allowing excellent quantification of this illicit drug.^39^

Whilst there are currently few examples in the SERS area, DoE is extensively utilised in other disciplines including: engineering, agriculture, social sciences as well as other analytical techniques. For example, in mass spectrometry based investigations for quantitative proteomics. Morris *et al*. (2010) used DoE for monitoring patterns of protein abundance in biological samples under various conditions and states. This enabled further understanding of the functioning of living organisms in search for early detection, diagnosis and prognosis of disease.^48^ Ultimately, the employment of DoE afforded the generation of reproducible and accurate results.

One of the more current areas where DoE is heavily exploited is in drug discovery of pharmaceuticals. Time and money are the major limitations holding back big drug discovery breakthroughs. DoE has widely been used in the optimisation and screening of experimental parameters. This allows key decisions to be made in the development of robust and reliable protocols in chemical synthesis, leading to optimal reaction conditions being identified in shorter periods of time.^49^ Moreover, in many lead discovery operations, assay development has been a major obstacle, consuming vast amounts of time and does not necessarily meet the desired assay quality parameters/signal window. DoE, in combination with high‐throughput technologies for drug efficacy, has emerged as a leading approach in overcoming this problem by effectively reducing the time taken without compromising on quality.^50^, ^51^

## Evolutionary computational approaches

One of the challenges in DoE is to navigate the experimental search space sufficiently, which is problematic as one cannot perform all possible experiments. Indeed, as the number of parameters that one wants to optimise increases linearly, the number of possible solutions increases exponentially. This is a so‐called NP hard (non‐deterministic polynomial‐time hard) problem and alternative search algorithms are needed.

Genetic algorithms (GAs) are heuristic search algorithms inspired by the Darwinian principle of evolution through natural selection.^52^ First proposed by Holland (1992), these computing techniques exploit a highly abstract version of evolutionary processes in order to solve problems efficiently for which there may be more than one potential solution; that is to say the central theme is that a good solution is appropriate rather than trying to find the best overall experimental conditions, as this is impossible (there simply is not enough time) to establish without conducting every single possible solution.^53^

One can consider a mountain range as an analogy for the experimental search space, where the height of the mountain (*z*‐axis) represents the assessment of the analytical result, and the *x*‐axis and *y*‐axis represent the parameters to be considered for optimisation. Here the higher the peak the better the experiment and the idea is to negotiate this landscape to reach the summit of the mountain range.

**Figure 3:**
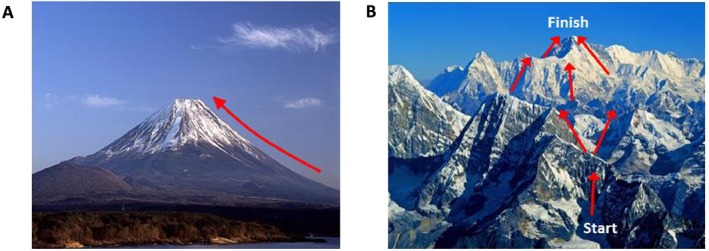
An illustration of the genetic algorithm (GA) approach to surface‐enhanced Raman scattering (SERS) optimisation using a mountain analogy. A GA reduces the number of steps taken in order to reach the highest fitness value through the evolution of solutions to find the highest peak. (A) Denotes a simple fitness landscape (e.g. Mount Fuji), where this may mean that only a single variable needs optimising. In this case, a GA approach is not necessarily required, and simple hill climbing algorithms would suffice. Whereas (B) highlights a more complex solution where multiple routes can be taken to reach the summit of the fitness landscape, i.e. multiple variables need to be optimised simultaneously, until the highest point is reached (here depicted by the Himalayas), and the application of GA may simplify the number of solutions to reach the optimum fitness value. The figures are available from the Creative Commons license agreement. For more details, see Mount Fuji image on Flickr https://www.flickr.com/photos/9177053@N05/4469232631/in/photostream/ and Himalayas image on Deviant art http://citizenfresh.deviantart.com/art/Himalaya‐Mountains‐1‐Nepal‐72353246

For relatively well‐behaved (some may say easy) optimisation search spaces, there may be a simple, obvious route for a given problem. This is illustrated by Mount Fuji in Fig. 3A, where the path to the summit is clear, and such a search may be possible using simple univariate statistics. By contrast, Fig. 3B depicts a more complex mountainous landscape, where the route taken to reach the summit is unclear whereby several paths could be taken. In such instances, the search is highly dimensional in nature and multivariate approaches are necessary. Implementation of a GA to this ‘Himalayan’ landscape could simplify and uncover the optimal route for reaching Everest.

**Figure 4:**
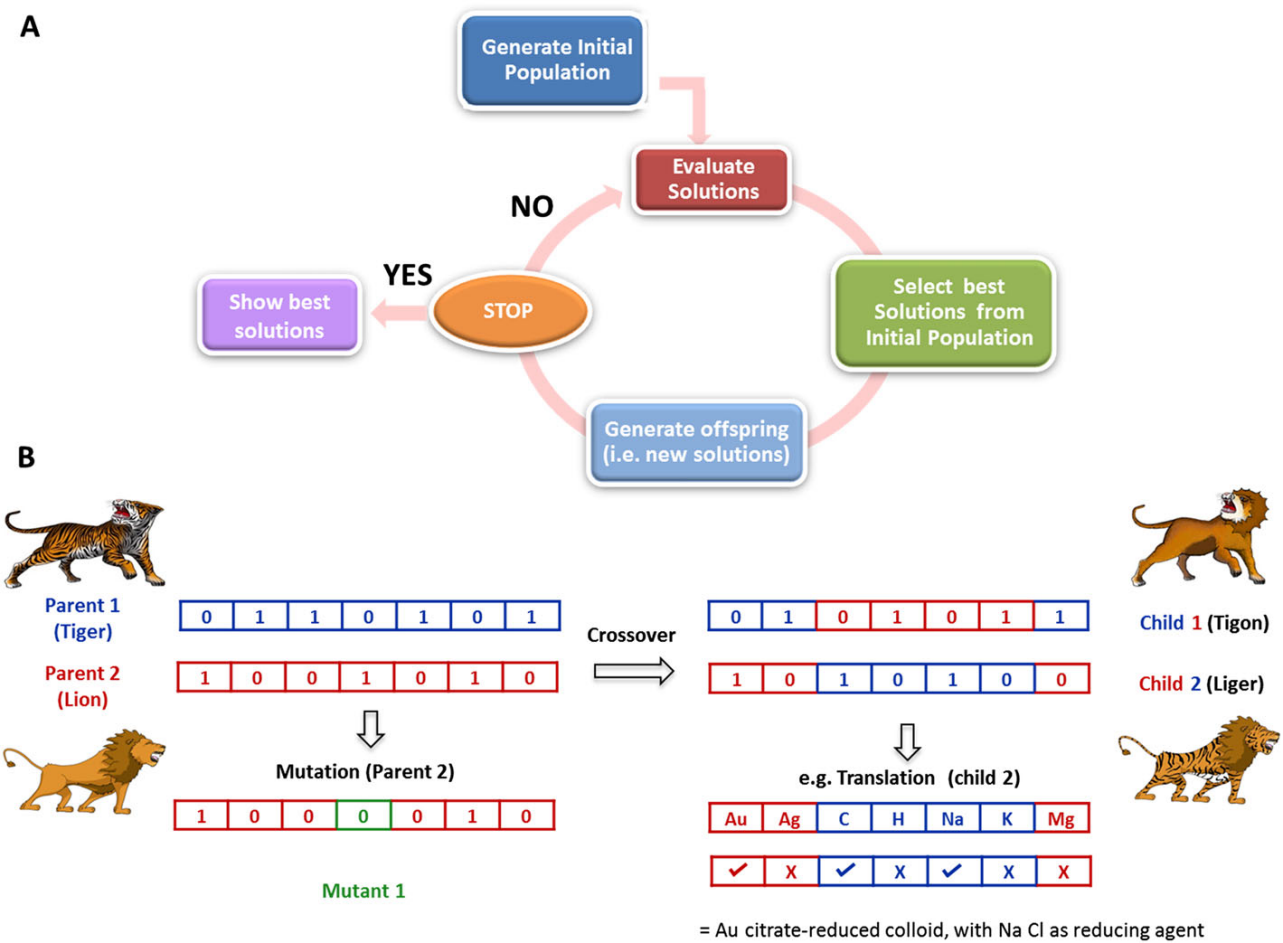
A schematic of the overall evolutionary approach that is used in experimental design. (A) Outlines a workflow of the genetic algorithm (GA) approach. The procedure continues to evolve until the population converges or when a maximum number of iterations is reached. (B) Denotes the methods used to generate a new population. These include offspring (in this example tigons and ligers) from the parents (a lion and a tiger). Mutations can also be generated and introduced from parents; e.g. mutant 1 is generated from a single‐point mutation to parent 2. This GA process needs translation in terms of surface‐enhanced Raman scattering optimisation: in the example provided, child 2 is translated as a solution where a Au citrate‐reduced colloid with NaCl as the reducing agent is used for surface‐enhanced Raman scattering.

Following on from the evolutionary analogy, GAs can be considered as a biological representation of DNA that is changed over a series of evolutions: each GA operates on a population of chromosomes (i.e. solutions to be tested), consisting of a number of genes (i.e. variables; one per parameter to be optimised). Each gene is binary encoded (0, 1), which can be thought of as an allele, where 0 = do not use this trait (e.g. a particular aggregating agent) and 1 = use this trait in the experiment. We note that there are richer encodings but these will not be discussed here.^53^ Implementation of this binary encoding allows a series of traits to be selected. In Fig. 4B, one can translate ‘Child 2’ (1, 0, 1, 0, 1, 0, 0) to represent a GA for use in SERS optimisation whereby the variables represent an experiment that uses Au citrate‐reduced colloid with NaCl as the reducing agent.

The overall GA process is depicted in Fig. 4A. The initial population is randomly generated and does not use any prior information that the analyst may have in terms of which parts of the search space may be better; this often requires some persuasion of the non‐cognoscenti. Next, these experiments are conducted in the laboratory. The SERS enhancement for each set of conditions is assessed and this allows one to rank these experiments according to their effectiveness at solving the problem (i.e. their fitness is calculated, with higher fitness being assigned to better experimental outcomes). A subset of this population is generated by means of ‘survival of the fittest’.

New experimental conditions now need to be generated (*vide infra*) to replenish the population and this process is analogous to biological reproduction (Fig. 4B). Chromosomes with the highest fitness values are selected as parents to recombine through crossover (mate) and produce the next population of children; alternatively, mutation may be used to modify the chromosome. In general the total population size remains constant throughout the evolutionary process.

The fitness of this new population is assessed. If the optimum solution is reached, the GA will stop, otherwise the evolutionary process will continue. The stop criterion is specific to the GA: common examples include a fixed number of generations, an observed convergence to a predetermined target (that is to say a solution has been generated that has a fitness greater than a particular value), or that after numerous generations the individual with the highest fitness value remains constant for several iterations.

Central to the GA is the way in which the population evolves over time. As well as asexual reproduction in which the children are merely clones of the parents, resulting in no diversification of the successive population, there are two main methods of producing new individuals from the previous population:

- Sexual reproduction, or crossover, occurs when two parents with high fitness values mate and swap alleles, generating two children chromosomes. Figure 4B illustrates this process where a ‘Tiger’ and ‘Lion’ are the parent chromosomes. A double crossover is shown, which generates two genetically different children – a ‘Tigon’ and a ‘Liger’ – in the hope of producing individuals with higher fitness values to that of its parents.
- Mutation can also occur whereby a single allele is randomly altered on a parent with a high fitness value, allowing for random divergence of the population. Figure 4B also shows a single mutation for Parent 2 (Lion), altering a random allele to the opposite gene (1 to 0).

There are many different factors to be considered when designing a GA. Examples include the following:

- Type of fitness function: This could be univariate *versus* multivariate assessment; SERS enhancement *versus* reproducibility or both combined in Pareto optimality.
- Population size.
- Rates of crossover and mutation operators – in general, mutation rates have a low probability of occurring, but their main purpose is to retain variation so that premature convergence does not occur.
- Evolutionary scheme that will be applied – some GAs use multiple populations that evolve independently that then cross fertilise.
- Finally the stop criterion (as discussed in the preceding section).

In summary, GAs are evolutionary computational‐based algorithms that are considered as powerful explanatory techniques. Whilst the primary aim here is to search the experimental landscape efficiently to generate a good SERS substrate and set of protocols for analyte detection/quantification, in other areas, GAs have been combined with chemometric approaches to effect variable selection and the interested reader is directed to.^54^, ^55^

## Application of evolutionary computational approaches to experimental optimisation

Genetic algorithms have proven to be highly efficient search models within a wide range of computer based science fields. He and Mort and Watanabe have successfully demonstrated GAs employment within communication network design and routing, ultimately minimising the path and increasing the reliability between routers.^56^, ^57^ Yao *et al*. optimised setup parameters for the simulation of a F1 race car, leading to enhanced performance and faster lap times.^58^ In addition, it would be remiss not to mention the numerous examples of the use of GAs in logistics and ‘the travelling salesman problem’. This scenario relates to the optimisation process involved in identifying the shortest route required to pass through each node (city) of a tour only once. An approach that has applications in the scheduling of shipping and routing of ships, as described by Al‐Hamad.^59^

More relevant examples have been described within the analytical chemistry field, demonstrating GAs effectiveness at deconvolving complex spectral datasets into simpler solutions. Tapp successfully established that different olive oils could be distinguished as a result of geographical origin by means of GA–LDA (linear discriminant analysis) approach using Fourier transform infrared spectroscopy data.^60^ Moreover, metabolomics has employed GAs because of the high dimensional multivariate data acquired. The field focuses on characterising small molecular metabolites involved in biological processes, using combinatorial techniques such as liquid chromatography–mass spectrometry or gas chromatography–mass spectrometry.^61^ Correa and Goodacre demonstrated that *Bacillus* species could be correctly identified and classified as a result of biomarker features that were selected by the GA from complex mass spectrometry data.^62^

To date, there are limited examples in the literature that exploit evolutionary computational approaches for the analysis of Raman and SERS data. Lavine and co‐workers successfully implemented a Raman spectroscopy‐GA approach in order to perform pattern recognition on several wood types, ultimately leading to their classification based on specific features, such as intensity at characteristic wavelengths.^63^

With respect to experimental optimisation, Jarvis *et al*. identified a key example in which SERS conditions for the detection of l‐cysteine were optimised by comparing the application of a multi‐objective evolutionary algorithm (MOEA) *versus* a full factorial design. The overall aim was to increase enhancement, as well as the reproducibility of SERS spectra. Two hundred and sixteen initial conditions were assessed using the later approach [(3 × colloidal substrate) × (6 × aggregating agent) × (3 × v/v colloid ratio) × (4 × aggregating agent concentration)], whereas the MOEA consisted of four generations, 20 experiments in each, composing of the variables previously stated. The evolutionary algorithm approach was shown to be superior, showcasing a 32% improvement in reproducibility and enhancement, using far less evaluations and thus being more cost effective.^47^ Finally, Levene and colleagues was able to decrease the limits of detection of propranolol (a *β*‐adrenergic blocker drug) 25‐fold lower than what was previously published. This substantial increase in sensitivity was achieved by developing a SERS–MOEA, based on Pareto optimality. The various experimental variables (metal type, aggregating agent, laser wavelength, etc.) investigated would have resulted in a full search consisting of 7785 experiments; however, enhanced experimental conditions were determined using only 4% of all the possible combinations.^64^

## Conclusion

From the literature, it is clear that there are many different approaches towards the optimisation of SERS experiments. However, as the SERS process is analyte dependent, with different chemical species having differing hydrophobicities, charges, sizes, as well as functional groups, there is no uniform optimisation protocol for one to follow. Notably, there is a compromise between intensity of SERS signals and reproducibility, and this is often determined by the researchers' experimental aim. For example, multiplexing experiments are not necessarily concerned with high levels of reproducibility, if the aim is just to detect more than one analyte at a time correctly. This optimisation approach would require intensity to be the most important factor, as similar levels of response for each analyte is required. However, if the aim was to quantify each of the analytes within the mixture, then reproducibility would become an increased concern. Evidently, identifying the ultimate goal prior to optimisation is key, as unavoidably there will be a trade‐off between the two objectives.

Throughout this review, we have highlighted examples of the different approaches that can be applied to enhance experimental results, such as DoE and GA. It is apparent that these models have been extensively used in active research fields yet seem to be currently limited within the optimisation of SERS systems. Perhaps a contributing factor in the opposition of DoE and GAs is the fear of statistics, with many researchers considering them to be a complication. In order to implement them successfully, there is a need for the researcher to have some understanding and appreciation of the statistical tests that underpin DoE and GAs before these approaches can become standard practice. We hope that the reader has found this review useful in this regard.
